# Burden of stroke in the Kingdom of Saudi Arabia: A soaring epidemic

**DOI:** 10.1016/j.jsps.2021.02.002

**Published:** 2021-02-12

**Authors:** Rehana Basri, Rakhi Issrani, Siew Hua Gan, Namdeo Prabhu, Mohammad Khursheed Alam

**Affiliations:** a Department of Internal Medicine, Neurology division, College of Medicine, Jouf University, Sakaka, Saudi Arabia; b Department of Preventive Dentistry, College of Dentistry, Jouf University, Sakaka, Saudi Arabia; c School of Pharmacy, Monash University Malaysia, Jalan Lagoon Selatan, Bandar Sunway, 47500 Subang Jaya, Selangor, Malaysia; d Department of Oral & Maxillofacial Surgery and Diagnostic Sciences, College of Dentistry, Jouf University, Sakaka, Saudi Arabia

**Keywords:** Stroke, Burden, Saudi Arabia, Cerebrovascular disease

## Abstract

Stroke is a key cerebrovascular disease that is related to high morbidity and mortality in the globe. The Kingdom of Saudi Arabia (KSA) is not an exception where stroke is fast developing into a serious challenge due to the high mortality rate. Additionally, stroke presents a tremendous economic burden and has a devastating effect on the quality of lives of individuals. The number of stroke cases are increasing yearly, thus posing a major challenge to the health care system. Therefore, it is crucial to implement primary and secondary prevention strategies in the KSA. Nevertheless, as compared with developed countries, information on the prevalence, socio-demographic properties and prevention of stroke remains scarce that could be attributed to the shortage of research conducted in this specified region. The review is written to address the various aspects of stroke in the KSA, based on current literatures search using PubMed, Scopus, Web of Science and Google Scholar databases, to identify studies published since inception to Dec 2020.

## 1 Introduction

Stroke leads to functional disability and long-standing neurologic injuries and is also associated with a high mortality rate worldwide. (Robert and Zamzami, 2014) Although the incidence of stroke is decreasing in many industrialized countries mainly due to better management of associated risk factors, the absolute number of stroke cases remain on an increasing trend due to aging population. (Lackland and Weber, 2015) The KSA faces a high burden of stroke due to an escalating incidence rate with mortality rate projected to be as nearly double by 2030. (Robert and Zamzami, 2014)

The largest country in the Middle East, the KSA makes up almost 80% of the Arabian Peninsula in terms of size in km (Lackland and Weber, 2015). (Robert and Zamzami, 2014) It has greater than 32 million population of which approximately 63% are the local Saudis, with the remaining consisting of working-age immigrants, originating mostly from Pakistan, India, Bangladesh and the Philippines. (Al-Senani et al., 2020) Although the KSA is considered as a high income country, with approximately USD 5,211 per capita gross domestic product (GDP), only 4.7% of its GDP is spent on health care which is much lower when compared to many other developed countries. (Robert and Zamzami, 2014, Al-Senani et al., 2020)

Based on the data reported by the World Health Organization, stroke is the second contributor to mortality in the KSA. (Al-Senani et al., 2020) The situation is compounded when stroke survivors are often left with serious physical and mental disabilities contributing to family, community and economic burdens to the country. (Robert and Zamzami, 2014) Despite these facts, previous studies have described a worryingly low level of stroke awareness in the population (Alzahrani et al., 2019, Alaqeel et al., 2014, Alhijji et al., 2018) that is worsened by the current lack of data on stroke in the KSA. The aim of this report is to provide an updated review of various aspects of stroke in the KSA based on the literatures available. PubMed, Scopus, Web of Science and Google Scholar databases were searched to identify studies published since inception to December 2020 with no language restriction placed.

## 2 Prevalence and incidence

If compared to other developed countries like the USA and UK, the incidence and prevalence of stroke is relatively low due to the predominance of the younger population in the KSA (Robert and Zamzami, 2014). There is a huge disparity in stroke incidence in the KSA as reported by the different studies **(**Table 1**)** (Al-Rajeh et al., 1993, Al-Rajeh et al., 1998, Ayoola et al., 2003, Almekhlaf, 2016, Alhazzani et al., 2018). It is plausible that the non-uniformity in the distribution of health care services in the KSA especially between the private and government sectors may play a role in contributing to the differences. (Alahmari and Paul, 2016) Although the data indicate a declining incidence at some point of time, in more recent studies, the stroke incidence in the KSA is on an upward trajectory (Alhazzani et al., 2018) with no decline in all mortalities and permanent disabilities seen especially in certain focused regions like Qunfuthah (Makkah province), Al Hasa (Eastern province), Jizan (Jizan Province) and Qassim (Jizan Province) (Akbar and Mushtaq, 2001) which are heavily populated and are more modernized.

**Table 1:** Incidence of stroke in the Kingdom of Saudi Arabia.

Authors and study year	Total population incidence rate per 100,000 person-years
Al-Rajeh et al. (1993) (Al-Rajeh et al., 1993)	43.80
Al-Rajeh et al. (1998) (Al-Rajeh et al., 1998)	29.80
Ayoola et al. (2003) (Ayoola et al., 2003)	15.90
Almekhlaf (2016) (Almekhlaf, 2016)	30.00
Alhazzani et al. (2018) (Alhazzani et al., 2018)	57.64

### 2.1 Influence of age and gender differences

Age is the most important risk factor for stroke. (Robert and Zamzami, 2014) Previous studies have shown that stroke tend to occur more frequently in the 61–70 age bracket and affect the younger age groups (20–30 years and 31–40 years ranges) to a lower extend. (Al-Jadid and Robert, 2010, Al-Eithan et al., 2011, Alotaibi et al., 2020, Yaqub et al., 1991) In fact, a study conducted by Al-Senani et al. (2020) stated that the mean age for the first incidence of stroke in Saudi Arabia is 63 years, as opposed to 69 years in the USA and about 70 years in the UK. (Al-Senani et al., 2020) It is plausible that the fact is contributed by the KSA population pyramid which is tilted towards a younger age band compared to the impact of lifestyle elements influencing risk for stroke. Research indicates a higher prevalence of stroke affecting male KSA patients than females (Al-Rajeh et al., 1993, Al-Jadid and Robert, 2010, Al-Eithan et al., 2011, Awada and Al-Rajeh, 1999, Memon et al., 2019, Sebai et al., 2001) similar to that for other countries like USA, Japan and Australia. The phenomenon may be contributed by the 1) protective hormonal effects in females or 2) higher prevalence of smokers among males and 3) more stressful lifestyles among males who are deemed as “the head of the family” as opposed to females (Appelros et al., 2009, Rasura et al., 2006).

### 2.2 Types of stroke

Strokes can be broadly classified into two major categories, *viz.*, ischaemic and haemorrhagic strokes. Ischaemic stroke is caused by interruption of the blood supply to a part of the brain resulting in a sudden loss of function whereas haemorrhagic stroke is related to an abnormal vascular structure or the rupture of blood vessel(s). (Donkor, 2018)

As shown in Table 2, ischemic stroke is the more predominant form affecting the KSA populations as compared with other types of strokes. As for the subtypes, non-lacunar variant (ischemic stroke) and intra-cerebral hemorrhage (hemorrhagic stroke) are more common. (Akbar and Mushtaq, 2001, Yaqub et al., 1991, Awada and Al-Rajeh, 1999, Al-Rajeh et al., 1993, Al-Rajeh et al., 1998, Ayoola et al., 2003, Al-Rajeh et al., 1991, Awada, 1994, El-sayed et al., 1999, Qari, 2000) According to the study done by Yaqub et al. (1991), the blood vessel most often affected is part of or the whole middle cerebral artery, constituting most of the arterial infarcts (52%). (Yaqub et al., 1991)

**Table 2:** Stroke subtypes in the Kingdom of Saudi Arabia.

Authors and study year	Stroke subtypes		Undefined (%)
	Ischemic (%) Non-L/L	Hemorrhagic (%) ICH/SAH	
Yacub *et al.* (1991) (Yaqub et al., 1991)	87.0 65.5/21.5	11.0 6.5/4.5	–
Al-Rajeh et al. (1991) (Al-Rajeh et al., 1991)	61.0 -/-	17.0 -/-	22.0
Al-Rajeh et al. (1993) (Al-Rajeh et al., 1993)	76.2 52/24.2	23.8 -/-	–
Awada (1994) (Awada, 1994)	58.5 51.0/7.5	41.5 32.5/9.0	–
Al-Rajeh et al. (1998) (Al-Rajeh et al., 1998)	69.0 45.0/24.0	18.0 16.6/1.4	13.0
Awada and Al-Rajeh (1999) (Awada and Al-Rajeh, 1999)	76.0 51.0/25.0	24.0 22.0/2.0	–
El-sayed et al. (1999) (El-sayed et al., 1999)	79.0 56.8/22.2	21.0 18.8/2.2	–
Qari (2000) (Qari, 2000)	77.0 -/-	20.5 -/-	–
Akbar and Mushtaq (2001) (Akbar and Mushtaq, 2001)	74.0 -/-	10.0 -/-	16.0
Ayoola et al. (2003) (Ayoola et al., 2003)	65.6 –	34.4 30.7/3.7	–

Non-L/L: non-lacunar/lacunar; ICH: intracerebral hemorrhage; SAH: subarachnoid hemorrhage.

### 2.3 Risk factors

The risk factors associated with stroke are usually classified into modifiable factors such as hypertension, diabetes mellitus, cardiovascular diseases, high blood cholesterol, atrial fibrillation sedentary lifestyle, obesity, smoking, alcohol consumption and non-modifiable factors like age and gender. (Alahmari and Paul, 2016, Donkor, 2018, Alharbi et al., 2019) It is important to note that these risk factors are important for both primary and secondary prevention of stroke. The other less documented risk factors may include the use of oral contraceptive, vasculitis, migraine, inflammatory processes, prothrombin activator inhibitor complex deficiency, hypotension, high C-reactive protein levels, sleep apnea syndrome, neurocysticercosis, chronic bronchitis, hyperuricemia, periodontal disease as well as infections by organisms such as *Helicobacter pylori, Legionella pneumophilia* and *Chlamydia pneumoniae*. (Donkor, 2018)

Arab populations have been reported to have similar lifestyles and diet to the Western and Oriental populations that may play a role in influencing the risk, type and survival after stroke. (Robert and Zamzami, 2014) The main predisposing factors identified for stroke among the population in Saudi are high blood pressure, followed by diabetes mellitus, cardiac disorders and cigarette smoking (Alhijji et al., 2018, Akbar and Mushtaq, 2001, Yaqub et al., 1991, Awada and Al-Rajeh, 1999, Donkor, 2018, Al-Rajeh et al., 1993, Al-Rajeh et al., 1998, Ayoola et al., 2003, Awada, 1994, El-sayed et al., 1999, Qari, 2000, Al-Rajeh et al., 1989, Al-Rajeh, 1994, Awada et al., 1996, Qutub, 2001) (Table 3**)**. Additionally, the combination of hypertension and diabetes mellitus is said to carry a higher risk for stroke especially among female patients. (Robert and Zamzami, 2014) Furthermore, maintaining a normal blood pressure and stopping cigarette are among possible lifestyle changes that can be incorporated to prevent stroke (Robert and Zamzami, 2014, Alharbi et al., 2019) in addition to incorporating anti-thrombotics to the treatment. A national survey on the diet the KSA indicated a high consumption of red meat and processed meat. (Moradi-Lakeh et al., 2017) Another interesting study indicated that the high consumption of olive oils among male Saudis seems to help lower the rate of cardiovascular disease. (Alkhaldy et al., 2019) Unfortunately, the risk factor management or lifestyle changes is poor in Saudi Arabia as confirmed by the study conducted by Robert et al. (2018) where only 23% of hypertensive patients were even aware that they have elevated blood pressures which co-exist with undiagnosed diabetes with micro- (28%) and macro-vascular complications (50%). (Robert et al., 2018)

**Table 3:** Frequency of risk factors in stroke cases reported in the Kingdom of Saudi Arabia.

Authors and study year	HBP (%)	DM (%)	Dyslipidemia (%)	Smoking (%)	TIA (%)	IHD (%)	AF (%)	RHD (%)	Not Specified (%)	FH (%)	Others (%)	None (%)
Al-Rajeh et al. (1989) (Al-Rajeh et al., 1989)	65.0	36.0	–	29.0	–	–	–	–	20.0	–	–	–
Yacub *et al.* (1991)**^a17^**	62.0H 41.0I	8.0H25.0I	–	–	–	10.0I	–	11.0I	–	–	–	–
Al-Rajeh et al. (1993) (Al-Rajeh et al., 1993)	56.0	42.0	–	6.0	9.0	–	–	–	33.0	–	3.0	–
Al-Rajeh (1994) (Al-Rajeh, 1994)	47.0	36.0	–	–	39.0	–	–	–	–	–	–	–
Awada (1994) (Awada, 1994)	32.0	16.0	–	26.0	6.0	–	–	–	17.0	–	1.0	–
Awada et al. (1996) (Awada et al., 1996)	64.0	23.0	–	–	20.0**^b^**	–	–	–	14.0	–	–	21
Al-Rajeh et al. (1998) (Al-Rajeh et al., 1998)	38.1	37.1	–	19.3	2.9	–	–	–	26.6	14.1	5.3	–
Awada and Al-Rajeh (1999) (Awada and Al-Rajeh, 1999)	52.0	41.0	–	10.4		17.0	10.0	–	–	–	3.0	–
El-sayed et al. (1999) (El-sayed et al., 1999)	24.9	11.6	–	1.8	2.1 2.1**^b^**	–	5.8	–	5.5	–	–	5.8 27.5**^c^**
Qari (2000) (Qari, 2000)	61.0	27.0	4.0	28.0	–	1.0	8.5	4.0	–	–	1.0	–
Qutub (2001) (Qutub, 2001)	80.0	72.0	36.0	–		–	–	–	–	–	–	–
Akbar and Mushtaq (2001) (Akbar and Mushtaq, 2001)	67.0	17.0	50.0	17.0	–	–	–	–	–	–	–	–
Ayoola et al. (2003) (Ayoola et al., 2003)	45.6	22.8	5.4	6.6	–	–	8.7	–	22.4	–	1.2	7.9
Alhijji B *et al.* (2018) (Alhijji et al., 2018)	24.9	11.6	–	1.8	2.1 2.1**^c^**	–	5.8	–	5.5	–	–	–
Alharbi MN *et al.* (2019) (Alharbi et al., 2019)	64.0	59.0	70.0	29.0	20.0	10.0	–	–	–	–	75.0	–

HBP: hypertension; DM: diabetes mellitus; TIA: previous history of transient ischemic stroke; IHD: ischemic heart disease; AF: atrial fibrillation; RHD: rheumatic heart disease; FH: family history of stroke.

a. Reported data on ‘H’ hemorrhagic and ‘I’ ischemic patients separately.

b. Previous history of stroke.

c. Data provided is only for hemorrhagic patients.

## 3 Health-related quality of life

Physical, cognitive and social functions are the main determinants of Health-Related Quality of Life (HRQoL) which is an imperative index for stroke outcome making its assessment is pivotal for a stroke survivor. (Hackett and Duncan, 2000, Suenkeler et al., 2002) Many factors such as gender, age and the need for daily living/disability undertakings as well as a low social support have been associated with low HRQoL values in stroke survivors. In fact, many reports have stated that stroke patients have lower Quality of Life (QoL) as compared to that of the general population in the first few years after a stroke episode, mainly in relation to the physical factors. (Hackett and Duncan, 2000, Suenkeler et al., 2002) A study conducted in the KSA confirmed that both functional condition and age play major roles in influencing HRQoL. (Gurcay et al., 2009) Overall, the QoL of stroke patients in the KSA is rather low as opposed to from some developed countries.

### 3.1 Length of hospital stay

Length of hospitalization (LoS) of stroke patients is generally influenced by factors such as age, gender, ethnicity, severity of stroke as well as the presence of any other medical comorbidities like hypertension, diabetes or cardiac illnesses that may unfavorably upset the outcome and LoS of stroke patients. (Appelros, 2007) A current hospital-based study in the KSA confirmed that the mean LoS for patients who received stroke rehabilitation program was 45 days. (Al-Eithan et al., 2011)

According to the study conducted by Al-Jadid and Robert (2010), there is a direct relationship between LoS and age where LoS for patients between 20 and 30 years old was only 36 days as compared to 53 days for those in the 71–80 age group. (Al-Eithan et al., 2011) Additionally, the LoS for Saudi males is longer than that for females reported to be attributed to poorer survival prospects of male stroke sufferers when compared to the females. (Di Carlo et al., 2003) In addition, males tend to be more susceptible to infections, injury and stress. (Hurn et al., 2005) Furthermore, females had lower overall risk for stroke severity, and subtype as well as cardiovascular risk factors when compared to males. (Di Carlo et al., 2003, De Reuck et al., 2008, Kapral et al., 2005)

With regards to ethnicity, the LoS of Saudis have been reported to be slightly higher (48 days) than that for non-Saudis (42 days) (Al-Eithan et al., 2011) thus supporting the fact that disparities in functional independence during admission to post-stroke rehabilitation and the mean daily functional improvement is contributed in part, to patients’ ethnicity. (Chiou-Tan et al., 2006) A recent study reported that the LoS of patients from the KSA with right hemiplegia/hemiparesis had a longer LoS value (47.3 days) as opposed to only 43.5 days for patients with left hemiplegia/hemiparesis. (Alotaibi et al., 2020) Right hemiplegia/hemiparesis occur due to stroke on the left side of the brain that leads to motor impairment which may explain the longer LoS. Patients suffering with stroke concurrently with other medical comorbidities tend to have a significantly higher LoS in patients with hemiplegia/hemiparesis since the medical complications adversely affect the functional outcome. (Alotaibi et al., 2020)

### 3.2 Healthcare services for stroke in the KSA

Extensive research is conducted worldwide on stroke care service planning to form national guidelines and recommendations on workforce as well as infrastructure needs to provide high-quality care in order to improve patient outcomes. (Al-Senani et al., 2020) The KSA Ministry of Health (MOH) is presently the chief source and financer of healthcare services that are supplemented by hospitals governed by other government agencies and private sector with most services found in the big cities. (Robert and Zamzami, 2014) Therefore, facilities are concentrated in big cities leading to inequity of services.

Rehabilitation programs and facilities which are central to modern health care provision are prioritized by the government and some private non-profit centers with high quality services accessible to all individuals with disabilities as well as in other populations in the country. However, most programs offer only physical, occupational, speech and hearing therapies besides prosthetic and orthotic services within the current state-of-the-art health care service system and infrastructure. (Al-Jadid, 2011) Based on the study conducted by Al-Senani et al. (2020), the population growth over a 10-year period was predicted to be at 62.7% from the beginning of the model, rising marginally to 64.4% after ten years. (Al-Senani et al., 2020) This means that although the KSA has a predominantly younger population, it still requires an augmented mandate on health care services in the future as the population ages.

Additionally, although there are more than 350 hospitals in the KSA, only one established active stroke center (at King Fahad Medical City, Riyadh, the KSA) and seven centers that provide thrombolysis for the stroke patients exist. Other than these, only two hospitals have a team dedicated to stroke patients which implements triaging pathways and a beeper system. (Qutub, 2001) A report by Al-Senani et al. (2019) on the present stroke services and staff availability within the KSA revealed that only 5% of stroke patients are admitted to acute stroke units situated in comprehensive stroke centers with specialist staff available. Therefore, the current staff numbers and services are insufficient to cater to the proposed growth in stroke cases. (Al-Senani et al., 2019) Thus, to offer acute and rehabilitation services with the use of the latest technologies, re-organization of existing staff and services are needed, in lieu with substantial investment in new rehabilitation medicine physicians or a team of rehabilitation professionals across several disciplines. (Al-Senani et al., 2019) All the factors above suggest that the current stroke care provision in the KSA is inadequate and remains below international recommendations. Thus, there is an urgent need to acquire a care pathway to provide advanced stroke services within the KSA.

## 4 Conclusion

Increase in stroke cases and mortality in the KSA is related to multiple factors like aging population, inequity of care across the KSA, low self-awareness and insufficient knowledge of stroke risk factors, causes and symptoms. This calls for an urgent need to develop a more competent and precise strategy for screening and diagnosis of stroke in the KSA along with having proper policies planning for proper primary prevention strategies, management programs and provision of health reserves. Additionally, there is a crucial need to increase public awareness especially among the younger population and those with lower education levels as to the risk factors causes and symptoms of stroke for better understanding of the negative impact of stroke on quality of life. The steps taken can help in disease prevention, also achievable by changing unhealthy lifestyles. Other than these initiatives, the development of well-equipped and trained health-care providers, a more coordinated multi-disciplinary approach as well as collaborative efforts among multidisciplinary teams are equally important.

## Disclosures

None
